# Supplementary material for: Theoretical approaches to process evaluations of complex interventions in health care: a systematic scoping review protocol
Source: Syst Rev. 2021 Oct 8;10:268. doi: 10.1186/s13643-021-01825-z (PMC8499466; doi:10.1186/s13643-021-01825-z)
Supplement: Supplementary file 1 — Additional file 1. PRISMA-P 2015 Checklist. [file 13643_2021_1825_MOESM1_ESM.docx]

Initial working definition: Process evaluation

Process evaluation is an integral part which runs parallel of the development and evaluation process of complex interventions (1). It is a freestanding methodology as well as closely linked to the methodology of outcome evaluation (2). Process evaluations aim to:

- assess (the quality of) implementation procedures (1-5);
- clarify causal mechanisms (1, 4) in relation to context, setting, professionals and patients (5);
- identify contextual factors influencing intervention implementation and intervention outcomes (1-5);
- explain the results of outcome evaluations (intervention failure, unexpected consequences/outcomes, intervention success) (1, 3-5);
- understand how an intervention can be optimized (4);
- contribute to an optimized description of the intervention, to create a model of change pathways of the intervention (3), and to distinguish between components of the intervention (2);
- achieve an understanding of the intervention that informs policy and practice (1).

Process evaluations may be conducted at different stages of the intervention development and evaluation process (e.g., feasibility testing, evaluation of the effectiveness of an intervention, post-evaluation scale-up). Thus the focus of a process evaluation will vary accordingly (1). Furthermore, the focus will differ between process evaluations with a focus on health care interventions and those that focus on implementation strategies for healthcare interventions (6).

**References**

1. Moore GF, Audrey S, Barker M, Bond L, Bonell C, Hardeman W, et al. Process evaluation of complex interventions - UK Medical Research Council (MRC) guidance. London: MRC Population Health Sciences Research Network; 2014 05/06/2020.

2. Oakley A, Strange V, Bonell C, Allen E, Stephenson J. Process evaluation in randomised controlled trials of complex interventions. BMJ. 2006;332(7538):413-6.

3. Baranowski T, Stables G. Process Evaluations of the 5-a-Day Projects. Health Education & Behavior. 2000;27(2):157-66.

4. Craig P, Dieppe P, Macintyre S, Michie S, Nazareth I, Petticrew M. Developing and evaluating complex interventions: the new Medical Research Council guidance. BMJ. 2008;337:a1655.

5. Grant A, Treweek S, Dreischulte T, Foy R, Guthrie B. Process evaluations for cluster-randomised trials of complex interventions: a proposed framework for design and reporting. Trials. 2013;14:15.

6. Hulscher ME, Laurant M, Grol R. Process evaluation of change interventions. In: Grol R, Wensing M, Eccles M, editors. Improving patient care: the implementation of change in clinical practice. London: Elsvier; 2005. p. 256-72.
