# Supplementary material for: Theoretical approaches to process evaluations of complex interventions in health care: a systematic scoping review protocol
Source: Syst Rev. 2021 Oct 8;10:268. doi: 10.1186/s13643-021-01825-z (PMC8499466; doi:10.1186/s13643-021-01825-z)
Supplement: Supplementary file 2 — Additional file 2. Initial working definition: Process evaluation. [file 13643_2021_1825_MOESM2_ESM.docx]

**Journal**

*BMC Systematic Reviews*

**Title**

Theoretical approaches to process evaluations of complex interventions in health care: a systematic scoping review protocol

**Authors**

Dr. Tina Quasdorf (TQ)^1, 2^

Prof. Dr. Lauren Clack (LC)^3^

Franziska Laporte Uribe (FLU), Ph.D. ^1, 2^

Prof. Dr. Daniela Holle (DH)^4^

Dr. Martin Berwig (MB)^1^

Daniel Purwins (DP)^1^

Dr. Marie-Therese Schultes^3^

Prof. Dr. Martina Roes (MR)^1, 2^

**Institutions**

^1^ DZNE, Deutsches Zentrum für Neurodegenerative Erkrankungen e.V. (DZNE), Witten, Germany

^2^ Department für Pflegewissenschaft, Fakultät für Gesundheit, Universität Witten/Herdecke, Germany

^3^ University of Zurich, Institute for Implementation Science in Health Care, Switzerland

^4^ Department für Pflegewissenschaft, Hochschule für Gesundheit, Bochum, Germany

**Corresponding author**

Tina Quasdorf

Deutsches Zentrum für Neurodegenerative Erkrankungen e.V. (DZNE), Witten

Stockumer Str. 12

58453 Witten, Germany

Tel.: +49 2302 926 237

Email: [tina.quasdorf@dzne.de](mailto:tina.quasdorf@dzne.de)

**Contact details**

Prof. Dr. Lauren Clack

University of Zurich, Institute for Implementation Science in Health Care

Universitätstrasse 84

8006 Zurich, Switzerland

Tel.: +41 79 750 13 96

Email: [Lauren.Clack@usz.ch](mailto:Lauren.Clack@usz.ch)

Franziska Laporte Uribe, Ph.D.

Deutsches Zentrum für Neurodegenerative Erkrankungen e.V. (DZNE), Witten

Stockumer Str. 12

58453 Witten, Germany

Tel.: +49 2302 926 237

Email: [franziska.laporte-uribe@dzne.de](mailto:franziska.laporte-uribe@dzne.de)

Prof. Dr. Daniela Holle

Department für Pflegewissenschaft, Hochschule für Gesundheit

Gesundheitscampus 6-8

44801 Bochum, Germany

Tel.: +49 234 77727 649

Email: [daniela.holle@hs-gesundheit.de](mailto:daniela.holle@hs-gesundheit.de)

Dr. Martin Berwig

Deutsches Zentrum für Neurodegenerative Erkrankungen e.V. (DZNE), Witten

Stockumer Str. 12

58453 Witten, Germany

Tel.: +49 2302 926 237

Email: [martin.berwig@dzne.de](mailto:martin.berwig@dzne.de)

Daniel Purwins

Deutsches Zentrum für Neurodegenerative Erkrankungen e.V. (DZNE), Witten

Stockumer Str. 12

58453 Witten, Germany

Tel.: +49 2302 926 237

Email: [daniel.purwins@dzne.de](mailto:daniel.purwins@dzne.de)

Dr. Marie-Therese Schultes

University of Zurich, Institute for Implementation Science in Health Care

Universitätstrasse 84

8006 Zurich, Switzerland

Tel.: +41 79 750 13 96

Email: [marie-therese.schultes@usz.ch](mailto:marie-therese.schultes@usz.ch)

Prof. Dr. Martina Roes

Deutsches Zentrum für Neurodegenerative Erkrankungen e.V. (DZNE), Witten

Stockumer Str. 12

58453 Witten

Germany

Tel.: +49 2302 926 237

Email: [martina.roes@dzne.de](mailto:martina.roes@dzne.de)

**Abstract** (max. 350 words)

*Background:* Complex interventions in health care are characterized by multiple interacting components as well as by numerous nonlinear interactions with the social systems within which they are being implemented. The process of developing, evaluating and implementing complex interventions is therefore challenging. Established guidance such as the MRC (Medical Research Council) framework for developing and evaluating complex interventions refers to process evaluations as an integral part of the development of complex evidence-based interventions. Even though the need for process evaluations is recognized, the realization of such approaches is challenging because methodological instruction is sparse, and the phenomenon of interest is complex. A number of theoretical approaches indicating how to conduct process evaluations of complex interventions in health care exist, but a systematic and comprehensive overview of these is missing. Thus, the objective of the systematic scoping review described herein is to provide an overview and analysis of theoretical approaches suitable for the planning and conducting of process evaluations.

*Methods:* The design and conduct of this review will follow the procedures of a systematic scoping review. The search strategy will be developed following the BeHEMoTh (Behaviour of interest; Health context; Exclusions; Models or Theories) template which has been conceptualized for structured reviews of theory. The systematic search of the Medline (via PubMed), CINAHL (via EBSCO) and PsycInfo (via EBSCO) electronic databases will be complemented by “hand searching” techniques. Study selection, data extraction, and data analysis will be performed by tandems of two researchers independently of each other. Divergent decisions and judgements between the two researchers will be discussed by the whole review team.

*Discussion:* The findings from this scoping review will provide an overview and comparison of theoretical approaches suitable for process evaluations of complex interventions in health care. The review results will support researchers in choosing the theoretical approach that best fits the respective focus of their process evaluation study.

*Systematic review registration:* This study has been registered with PROSPERO (International Prospective Register of Systematic Reviews) under registration number CRD42020211732.

**Keywords (**3-10)

process evaluation, frameworks, models, theory, complex intervention, implementation, health care, systematic scoping review

**Number of words:** 3590

**Background**

Efforts to improve healthcare delivery and outcomes often require complex interventions. Complex interventions were originally defined as interventions with multiple interacting components (1, 2), indicating that complexity is particularly linked to the intervention itself. Ongoing debates about which characteristics constitute complex interventions (3-5) have complemented this original definition with the awareness that complexity is given “…primarily due to the social systems within which these actions occur, the contextually contingent nature of impacts, and the agency of the groups and individuals whose behaviours they aim to influence.”(3)

Due to this changing definition, it is increasingly recommended to integrate process evaluations in the development and evaluation of complex evidence-based interventions to assess implementation aspects and to gain a better understanding of causal mechanisms and contextual factors associated with variation in outcomes (1, 6-8). Process evaluations can especially help to distinguish between those interventions that are inherently faulty (failure of intervention) and those interventions that are delivered inaccurately (implementation failure) (9). Thus, they can prevent a so-called type III error, which refers to the question of whether the intervention actually has been delivered or if there is in fact something measured that does not exist (10).

The MRC (Medical Research Council) framework for developing and evaluating complex interventions provides a well-established guide for the development of complex interventions in healthcare (1, 2). The revised version from 2008 (1, 11) for the first time took into account that process evaluations are highly recommendable to understand processes and to obtain explanations if interventions fail or have unanticipated consequences (1). Nevertheless, the 2008 framework provided only sparse information on how to integrate and conduct process evaluations. Process evaluation is described as being “nested within a trial” that in the first place focuses on outcome evaluation (11).

In recent years, methodological aspects of process evaluations have been increasingly discussed; thus, the current MRC provides comprehensive guidance for process evaluations of complex interventions (6, 12). This guidance refers to 1) *implementation*, 2) *mechanisms of impact*, and 3) *context* as key aspects to be examined (6, 12). Furthermore, it “provides researchers, practitioners, funders, journal editors and policy-makers with guidance in planning, designing, conducting and appraising process evaluations of complex interventions” (12, p. 9). It comprises process evaluation theory as well as practical information (12).

Regardless of the scientific discourse in the context of the MRC framework, the concept of process evaluation is not new to the field of health care research; it had already emerged by the 1960s (13). Suchmann (14), as one of the first to emphasize the need to evaluate health interventions/programmes in relation to their practical setting, identified 1) *the attributes of the programme*, 2) *the population exposed to the programme*, 3) *the situational context in which the programme takes place*, and 4) *the different kinds of effects* produced by the programme as obligatory domains for “making sense” of the evaluative findings. Although these basic assumptions of Suchmann (14) remain relevant for the context of process evaluation, a wide range of developments have produced a diverse research field with many underlying concepts.

Commonly quoted concepts are recruitment (of cluster/of individuals), maintenance, context, resources, implementation, reach (of individuals), barriers, exposure, initial use, continued use, contamination, dose delivered, dose received, fidelity (degree to which an intervention is implemented in accordance with the original protocol), delivery (to clusters/to individuals), response (of clusters/of individuals), effectiveness, unintended consequences, theory, efficacy, and adoption (13, 15-17). Many of these concepts are also closely linked to the broad field of implementation research (18). Thus, the underlying theoretical allocation and a clear distinction of these concepts is challenging.

The existing theoretical approaches relevant for process evaluations set varying priorities and organize their concepts in different ways (e.g. 19). In addition, concepts termed identically in different theoretical approaches are not always defined in the same way, and they are not always selective between the different theoretical approaches. The MRC guidance for process evaluation of complex interventions (12) presents “an overview of a number of core frameworks” (12, p. 18) relevant to process evaluation, but the authors explicitly state that they “make no claims to exhaustiveness” (12, p. 18). While the theoretical approaches considered in the MRC guidance for process evaluation include many of the concepts mentioned above, the guidance does not provide a thorough analysis or comparison of the concepts the theoretical approaches refer to. Therefore, it remains unclear why the proposed theoretical approaches were selected and if there may be other theoretical approaches of relevance. This complicates the suggestion of the MRC guidance to combine concepts from different theoretical approaches for the development and conduct of process evaluations (12). However, given the variety of theoretical approaches that are of relevance for process evaluations, it remains challenging to select and combine theoretical approaches or single concepts that fit the requirements and aims of a specific process evaluation approach.

Therefore, we will perform a comprehensive and systematic scoping review and analysis of the theoretical approaches suitable for process evaluations of complex interventions in health care. For this purpose, we understand theoretical approaches as interrelated propositions that intend to explain and predict general sets of phenomena (20). Accordingly theoretical approaches help to structure our knowledge, understanding, explanation, and prediction of the world and they allow to generate new research hypotheses as well (20-22). In the context of process evaluation, a theoretical foundation is essential to better understand and systematically investigate the black box of implementing complex interventions in complex social settings (22). At the same time theoretical approaches are not uniform, but might be e.g. implicit or explicit, descriptive, explanatory or predictive (22) or address high, middle or low abstraction level (21). Moreover, a range of heterogenous terms is being used in order to consider theoretical aspects in research projects and real life, e.g. theoretical/conceptual model, (theoretical/conceptual) framework, theoretical concepts (21, 22) These terms are often not clearly distinguished and used in an interchangeable way (20, 21). Based on the above-noted explanations we will therefore consider any kind of theoretical approach suitable for process evaluation in order to achieve a comprehensive overview.

The review results will further support researchers in considering, selecting and combining the available theoretical approaches (or just single concepts) and thus promote tailored and theory-informed process evaluation approaches.

*Objectives*

The following study objectives were defined:

- To identify theoretical approaches suitable for process evaluations of complex interventions in health care;
- To describe, analyse and compare the identified theoretical approaches and single concepts that these theoretical approaches comprise of.

**Methods**

We will perform a review of theoretical approaches. This kind of review differs substantially from a review of empirical data. Methodological issues related to such approaches have been rarely discussed in the literature (23), and they are not taken into account in overviews of review types (24, 25). We will therefore consider the methodological issues related to this review carefully and ensure its transparency and comprehensiveness. The development and reporting of the review will follow the PRISMA (**P**referred **R**eporting **I**tems for **S**ystematic Reviews and **M**eta-**A**nalyses) extension for Scoping Reviews (PRISMA-ScR). The PRISMA extension, PRISMA-P (26) (see Additional file 1) has been used for the preparation of this review protocol.

Important protocol amendments will be reported with the review results.

*Searches*

We will use the BeHEMoTh (Behaviour of interest; Health context; Exclusions; Models or Theories) template only to develop our search strategy. This template has been recently conceived to inform the development of procedures for the structured review and identification of theory (27). It refers to the following categories:

- Be – Behaviour of interest 🡪 ways in which the population interacts with the health context;
- H – Health context of interest;
- E – Exclusions 🡪 to exclude, e.g., non-theoretical/technical models/frameworks; and
- MoTh – Models or Theories 🡪 generic terms for models/frameworks, as well as named frameworks/models, if appropriate.

Table 1 gives an overview of the initial search terms which will be refined during the search process.

Table 1: Initial search terms based on the BeHEMoTh framework (27)

| Be: Behaviour of interest | H: Health context | E: Exclusions | MoTh: Models or Theories |
| --- | --- | --- | --- |
| “process evaluation*” [TiAb]  “formative evaluation*” [TiAb]  “qualitative evaluation*” [TiAb]  “implementation evaluation^*^” [TiAb] | complex interventions in health care | non-theoretical models, e.g., statistical models  **both dimensions considered for in-/exclusion criteria but NOT for search strategy** | model*[TiAb]  frame*[TiAb]  concept*[TiAb]  guid*[TiAb]  theor* [TiAb] |

[TiAb] = search for this term in titles and abstracts

Based on the final search strategy, systematic electronic searches of the following databases relevant to health care research will be performed: Medline (via PubMed), CINAHL and PsycInfo (both via EBSCO).

Furthermore, iterative, pragmatic approaches, in addition to keyword searches alone, are recommended as an effective method for qualitative reviews (28, 29) and seem to suit the needs of reviews of theory as well.

Thus, following the iterative approach, the systematic search will be complemented by “hand-searching” techniques such as expert consultations, trawls through specific journals, and backward as well as forward citation tracking, which is frequently recommended as being especially valuable for reviews of theory (23).

The development and application of the search strategy will be continuously discussed by the project team, which comprises all the authors of this study protocol.

*Study criteria*

The search results will be screened according to defined inclusion criteria and thereby resulting exclusion criteria. An initial set of inclusion criteria is outlined in table 2.

Table 2: Outline of initial inclusion criteria of the review

| Criterion | Definition |
| --- | --- |
| Process evaluation | Publications that focus on a theoretical approach that has been developed explicitly for process evaluations of complex interventions in health care (e.g., methodological articles; theoretical debates; empirical articles developing/testing theoretical approaches for process evaluation)  OR  publications reporting process evaluations that apply an existing theoretical approach 🡪 search for the original publication of the applied theoretical approach will be performed  OR  publications reporting studies that are not labelled as “process evaluation”, but that actually meet the criteria of a process evaluation (see working definition in additional file 2) and that apply an existing theoretical approach 🡪 search for the original publication of the applied theoretical approach will be performed |
| Health context | any field of health care |
| Theoretical approach | key publication with thorough description of a the theoretical approach must be available (including information on, e.g., domains/dimensions/concepts/processes/strategies of the theoretical approach) |
| Type of publication | any type of publication that focuses on the development or application of theoretical approaches for process evaluation in health care (e.g., (reviews of) methodological articles; theoretical debates; empirical articles developing/testing/applying theoretical approaches for process evaluation) |
| Other | Languages: English, German |
|  | Year of publication: no limitation |
|  |  |

For the purpose of our review, precise criteria definitions based on established frameworks, such as PICO (30) or its equivalent for reviews of qualitative studies, SPIDER (28), developed in the context of health research are not suitable, since relevant theoretical approaches possibly do not originate from the specific field of process evaluation or from health care research (23). Further, one of the key challenges in this review will be to define which criteria constitute a “process evaluation” since a consistent terminology and definition is missing. Thus, from the beginning of the review process we have discussed within the review team which criteria could define “process evaluation”. We started out with a literature-based working definition (additional file 2), which we will continuously develop throughout the review process in iterative loops. The in- and exclusion criteria will be adapted accordingly. Following the iterative approach described above, amendments and specifications of the initial set of criteria will be discussed and decided throughout the review process. All decisions and amendments are being documented in a study manual, which will be provided in the final review paper to ensure transparency. .

*Data management*

Data management will be organized using the EPPI-Reviewer4 software (31), which has been developed for managing and analysing data in all types of systematic reviews, such as meta-analysis, framework synthesis and thematic synthesis (32). Features of the software support reference management, study classification, data extraction, and synthesis. The software is recommended by the Cochrane Collaboration (33).

*Selection process*

Duplicates will be removed before starting the selection process. This step, as well as the other steps of the selection process, will be supported by the EPPI-Reviewer4 software.

Based on the final set of inclusion and exclusion criteria, all titles and abstracts will be screened independently by each researcher paired up in teams of two (tandems) (all authors of this study protocol). Full texts will be obtained and assessed where the inclusion criteria seem to be met but where ratings for the title and abstract screenings differ between reviewers and no consensus can be achieved. Cases that remain unclear after a full text assessment will be discussed within the tandems and agreed upon between both reviewers. In case of non-agreement within one tandem, the publication will be discussed within the project team for a final decision.

In- and exclusion of publications reporting studies that are not labelled as “process evaluations”, but that actually meet the criteria of a process evaluation and that apply a theoretical approach will be thoroughly discussed within the project team. This discussion will be based on a working definition of “process evaluation” that will be developed consecutively throughout the review process.

Publications describing the same framework will be consolidated before data extraction since the framework/model is the unit of interest and not the single report (33). Different versions and developments of frameworks/models will be distinguished.

*Data extraction strategy*

The EPPI-Reviewer4 software (32) will be used for data extraction. This software allows flexible coding schemes for classifying studies with regard to eligibility criteria and for capturing detailed information from the included references. We will extract data based on the following coding categories: author, year, name of theoretical approaches, scope, process of development, historical roots/theoretical basis, dimensions and constructs included in the theoretical approaches, and guidance on the utilization of the theoretical approaches.

The data extraction will again be performed independently by each researcher paired up in tandems . The EPPI-Reviewer4 software (32) allows the classification of theoretical approaches by multiple users, the comparison of results, and the documentation of the process of agreeing upon final decisions.

*Data synthesis and presentation*

The identified theoretical approaches will be presented in a table format. These tables will provide an overview and content-wise sorting of the identified theoretical approaches. The tables will also present the data extracted with regard to each of the aforementioned categories (see data extraction). A main focus will be the display and allocation of the single concepts based on the identified theoretical approaches. They will also be presented in a table. However, since concepts used in the context of process evaluation often overlap and are inconsistently defined, we will also consider divergent definitions and meanings.

The review results will be published in a peer-reviewed journal and presented at relevant conferences.

**Discussion**

The relevance of process evaluations is being increasingly discussed in the scientific discourse on the development and evaluation of complex interventions and is now widely recognized (1, 6, 8, 15, 16). Although an increasing number of process evaluations are being conducted, these approaches are often not well founded on theory since there is little guidance available on how to derive concrete process evaluation procedures from theoretical allocation (16). Frequently, this leads to arbitrary methodological procedures and subsequently to low-quality results. Furthermore, the phenomenon investigated is very demanding, since the focus is not only on the intervention to be developed but also on the implementation, the mechanisms of impact, and above all on the context (1, 6), which makes it necessary to consider complex social systems (3). A theoretical foundation is therefore essential for the planning and realization of process evaluations. The results of this review will provide a systematic and critical overview and comparison of existing theories for process evaluations and thus facilitate the targeted selection of suitable theoretical approaches for the respective process evaluation approach. The review will build upon and further contribute to the scientific discourse on process evaluations that has been inspired by the MRC and its guidance on process evaluation.

From a methodological point of view, there will be some challenges related to this review since little literature exists with regard to literature reviews aiming at identifying theory. However, we expect that we will achieve high-quality results with the methodological approach described in this protocol, which is based on the guidelines for conducting systematic scoping reviews and the demonstrated iterative, inductive and reflexive approaches of qualitative research.

**List of Abbreviations**

BeHEMoTh Behaviour of interest; Health context; Exclusions; Models or Theories

MRC Medical Research Council

PRISMA Preferred Reporting Items for Systematic Reviews and Meta-Analyses

PRISMA-P PRISMA for Protocols

PRISMA-ScR PRISMA Extension for Scoping Reviews

PROSPERO Prospective Register of Systematic Reviews

**Declarations**

*Ethics approval and consent to participate*

Not applicable

*Consent for publication*

Not applicable

*Availability of data and materials*

Not applicable

*Competing interests*

The authors declare that they have no competing interests.

*Funding*

No third-party funding has been or will be received for this study; the study will be funded by the German Center for Neurodegenerative Diseases e.V. (DZNE) site Witten.

*Authors' contributions*

Contributed substantially to conception and design of the review: TQ, LC, FLU, DH, MR

Drafted the manuscript: TQ

Revised the manuscript critically for important intellectual content: TQ, LC, FLU, DH, MR

Provided final approval of the version to be published: TQ, LC, FLU, DH, MR

Agreed to be accountable for all aspects of the work with respect to ensuring that questions related to the accuracy or integrity of any part of the work are appropriately investigated and resolved: TQ, LC, FLU, DH, MR

Guarantor of the paper: TQ

*Acknowledgements*

Not applicable

**References**

1. Craig P, Dieppe P, Macintyre S, Michie S, Nazareth I, Petticrew M. Developing and evaluating complex interventions: the new Medical Research Council guidance. BMJ. 2008;337:a1655.

2. Campbell M, Fitzpatrick R, Haines A, Kinmonth AL, Sandercock P, Spiegelhalter D, et al. Framework for design and evaluation of complex interventions to improve health. BMJ. 2000;321(7262):694-6.

3. Moore GF, Evans RE, Hawkins J, Littlecott H, Melendez-Torres GJ, Bonell C, et al. From complex social interventions to interventions in complex social systems: Future directions and unresolved questions for intervention development and evaluation. Evaluation. 2019;25(1):23-45.

4. Hawe P, Shiell A, Riley T. Theorising Interventions as Events in Systems. American Journal of Community Psychology. 2009;43(3-4):267-76.

5. Shiell A, Hawe P, Gold L. Complex interventions or complex systems? Implications for health economic evaluation. BMJ. 2008;336(7656):1281-3.

6. Moore GF, Audrey S, Barker M, Bond L, Bonell C, Hardeman W, et al. Process evaluation of complex interventions: Medical Research Council guidance. BMJ. 2015;350.

7. Vernooij-Dassen M, Moniz-Cook E. Raising the standard of applied dementia care research: addressing the implementation error. Aging & Mental Health. 2014;18(7):809-14.

8. Oakley A, Strange V, Bonell C, Allen E, Stephenson J. Process evaluation in randomised controlled trials of complex interventions. BMJ. 2006;332(7538):413-6.

9. Rychetnik L, Frommer M, Hawe P, Shiell A. Criteria for evaluating evidence on public health interventions. J Epidemiol Community Health. 2002;56(2):119-27.

10. Dobson D, Cook TJ. Avoiding type III error in program evaluation: Results from a field experiment. Evaluation and Program Planning. 1980;3(4):269-76.

11. Craig P, Dieppe P, Macintyre S, Michie S, Nazareth I, Petticrew M. Developing and evaluating complex interventions: new guidance. London: MedicalResearchCouncil; 2008.

12. Moore GF, Audrey S, Barker M, Bond L, Bonell C, Hardeman W, et al. Process evaluation of complex interventions - UK Medical Research Council (MRC) guidance. London: MRC Population Health Sciences Research Network; 2014 05/06/2020.

13. Linnan L, Steckler A. Process Evaluation for Public Health Interventions and Research:An Overview. In: Steckler A, Linnan L, editors. Process Evaluation for Public Health Interventions and Research. New York: John Wiley & Sons Inc; 2002.

14. Suchmann E. Evaluative Research: Principles and Practice in Public Service and Social Action Programms: Russell Sage Foundation; 1967.

15. Baranowski T, Stables G. Process Evaluations of the 5-a-Day Projects. Health Education & Behavior. 2000;27(2):157-66.

16. Grant A, Treweek S, Dreischulte T, Foy R, Guthrie B. Process evaluations for cluster-randomised trials of complex interventions: a proposed framework for design and reporting. Trials. 2013;14:15.

17. Glasgow RE, Vogt TM, Boles SM. Evaluating the public health impact of health promotion interventions: the RE-AIM framework. American journal of public health. 1999;89(9):1322-7.

18. Rabin BA, Brownson RC, Haire-Joshu D, Kreuter MW, Weaver NL. A glossary for dissemination and implementation research in health. Journal of public health management and practice : JPHMP. 2008;14(2):117-23.

19. Leontjevas R, Gerritsen DL, Koopmans RT, Smalbrugge M, Vernooij-Dassen MJ. Process evaluation to explore internal and external validity of the "Act in Case of Depression" care program in nursing homes. J Am Med Dir Assoc. 2012;13(5):488.e1-8.

20. Carpiano RM, Daley DM. A guide and glossary on post-positivist theory building for population health. Journal of epidemiology and community health. 2006;60(7):564-70.

21. Nilsen P. Making sense of implementation theories, models and frameworks. Implementation Science. 2015;10(1):53.

22. Rycroft-Malone J, Bucknall T. Theory, frameworks, and models - Laying down the groundwork. In: Rycroft-Malone J, Bucknall T, editors. Models and Frameworks for Implementing Evidence-Based Practice. 1 ed: John Wiley and Sons Ltd; 2010.

23. Campbell M, Egan M, Lorenc T, Bond L, Popham F, Fenton C, et al. Considering methodological options for reviews of theory: illustrated by a review of theories linking income and health. Systematic reviews. 2014;3(1):114.

24. Grant MJ, Booth A. A typology of reviews: an analysis of 14 review types and associated methodologies. Health information and libraries journal. 2009;26(2):91-108.

25. Booth A, Sutton A, Papaioannou D. Systematic Approaches to a Successful Literature Review. 2 ed. London: SAGE Publications Ltd; 2016.

26. Moher D, Shamseer L, Clarke M, Ghersi D, Liberati A, Petticrew M, et al. Preferred reporting items for systematic review and meta-analysis protocols (PRISMA-P) 2015 statement. Systematic reviews. 2015;4:1.

27. Booth A, Carroll C. Systematic searching for theory to inform systematic reviews: is it feasible? Is it desirable? Health Information & Libraries Journal. 2015;32(3):220-35.

28. Cooke A, Smith D, Booth A. Beyond PICO: The SPIDER Tool for Qualitative Evidence Synthesis. Qualitative Health Research. 2012;22(10):1435-43.

29. Thomas J, Harden A. Methods for the thematic synthesis of qualitative research in systematic reviews. BMC Medical Research Methodology. 2008;8(1):45.

30. Thomas J, Kneale D, McKenzie JE, Brennan SE, Bhaumik S. Chapter 2: Determining the scope of the review and the questions it will address. In: Higgins JP, Thomas J, Chandler J, Cumpston M, Li T, Page MJ, et al., editors. Cochrane Handbook for Systematic Reviews of Interventions. 2 ed. Chichester (UK): John Wiley & Sons; 2019. p. 13–32.

31. Thomas J, Graziosi S, Brunton J, Ghouze Z, O'Driscoll P, Bond M. EPPI-Reviewer: advanced software for systematic reviews, maps and evidence synthesis. EPPI-Centre Software. London: UCL Social Research Institute; 2020.

32. EPPI-Reviewer 4 - Software for research synthesis: User Manual. London: Social Science Research Unit, UCL Institute of Education; 2018. Available from: <http://eppi.ioe.ac.uk/CMS/Portals/35/ER4_8_0%20user%20manual.pdf>.

33. Lefebvre C, Glanville J, Briscoe S, Littlewood A, Marshall C, Metzendorf M-I, et al. Chapter 4: Searching for and selecting studies. In: Higgins JP, Thomas J, Chandler J, Cumpston M, Li T, Page MJ, et al., editors. Cochrane Handbook for Systematic Reviews of Interventions. 2 ed. Chichester (UK): John Wiley & Sons; 2019. p. 67-108.

**Additional files**

*Additional file 1*

- Word document (docx)
- Title of data: PRISMA-P 2015 Checklist

*Additional file 2*

- Word document (docx)
- Title of data: Initial working definition: Process evaluation
